# Effect of Encapsulation Techniques on Aroma Retention of *Pistacia terebinthus* L. Fruit Oil: Spray Drying, Spray Freeze Drying, and Freeze Drying

**DOI:** 10.3390/foods12173244

**Published:** 2023-08-29

**Authors:** Delal Meryem Yaman, Derya Koçak Yanık, Aysel Elik Demir, Hicran Uzun Karka, Gamze Güçlü, Serkan Selli, Haşim Kelebek, Fahrettin Göğüş

**Affiliations:** 1 Engineering Faculty, Food Engineering Department, Gaziantep University, 27310 Gaziantep, Turkey; deelal12@hotmail.com (D.M.Y.); derya.kocakyanik@ogu.edu.tr (D.K.Y.); ayselelik@tarsus.edu.tr (A.E.D.); 2 Department of Food Engineering, Faculty of Agriculture, Eskişehir Osmangazi University, Eskişehir 26040, Turkey; 3 Department of Food Technology, Vocational School of Technical Sciences Vocational School of Technical Sciences at Mersin Tarsus Organized Industrial Zone, Tarsus University, Mersin 33000, Turkey; 4 Department of Food Processing, Vocational School of Technical Sciences of Gaziantep University, 27310 Gaziantep, Turkey; hicranuzun@gantep.edu.tr; 5 Department of Food Engineering, Faculty of Agriculture, Çukurova University, Adana 01380, Turkey; gguclu@cu.edu.tr (G.G.); sselli@cu.edu.tr (S.S.); 6 Faculty of Engineering, Department of Food Engineering, Adana Alparslan Türkeş Science and Technology, Adana 01250, Turkey; hkelebek@atu.edu.tr

**Keywords:** aroma, encapsulation, spray drying, spray freeze-drying, fruit oil

## Abstract

The primary aim of this investigation was to assess the impact of varying the ratio of gum arabic to maltodextrin and employing diverse encapsulation techniques on the properties of the powdered substance and the capacity to retain the aromatic attributes of terebinth fruit oil. Distinct ratios of gum arabic to maltodextrin (75:25, 50:50, and 25:75) were employed to fabricate oil-in-water emulsions. The utmost stability of the emulsion was realized at a gum arabic to maltodextrin ratio of 75:25, characterized by a minimal creaming index and an even and small-scale dispersion. The encapsulation techniques employed included spray drying (SD), spray freeze-drying (SFD), and freeze-drying (FD). These methodologies were compared based on encapsulation efficiency, desiccation yield, powder attributes, and the capacity to retain aroma. The encapsulation efficiencies were notably higher (>90%) in SD, particularly with the application of an ultrasonic nozzle and a two-fluidized nozzle (2FN), in contrast to those obtained through SFD and FD. Notably, SD employing an ultrasonic nozzle exhibited superior preservation of volatiles (73.19%) compared to FD (24.45%), SD-2FN (62.34%), and SFD (14.23%). Among the various components, α-pinene and linalool stood out with near-perfect retention rates, close to 100%.

## 1. Introduction

*Pistacia terebinthus* L. (Anacardiaceae) is a pistacia species widely distributed in the Mediterranean region, ranging from Morocco and Portugal through Greece, Turkey, Syria, and Asia [1,2]. The small spherical terebinth fruits are dark greenish in color in the ripe stage. This fruit is traditionally used in various regions of the world for different purposes such as appetizers, snack food, food ingredients, coffee, cooking oil, soap making, folk medicine, etc. Additionally, it is used in baking traditional country bread and in producing traditional soap known as bıttım soap in Turkey [3,4]. The fruit of this plant is rich in oil content (58–60%) and aromatic components [5]. PTFO, like many peanut oils, is a very valuable oil with its high unsaturated fatty acid content. It has a unique fatty acid composition in which oleic acid is the highest fatty acid (around 52.3%). Moreover, its oleic acid is mainly located in the sn-2 position of the glycerol backbone. This makes it very similar to olive oil [5]. Therefore, it can be considered a beneficial oil for health. Gogus et al. [6] reported that α-pinene, limonene, γ- cadinene, β-pinene, and β-caryophyllene are the main volatile compounds in fresh whole terebinth fruits. Amanpour et al. [7] investigated the main aroma compounds of terebinth fruits and reported that β-myrcene and α-pinene were the most powerful key aroma compounds. Also, α-pinene is the major volatile compound of the pistachio hull, with 54.4% [8]. Limonene and α-Pinene are also important aroma compounds in pistachio nuts [9,10]. Therefore, *Pistacia terebinthus* fruit oil (PTFO) can be considered as an alternative with a similar scent to the pistachio nut aroma.

Aroma compounds in food materials are influenced by various environmental factors, such as temperature, soil type, sunlight, and availability of water [11]. They contribute significantly to the aroma of food products and remarkably influence consumer perception and acceptability [12]. However, they are highly volatile; they are likely to be easily lost at different stages of food processing. In this regard, it is common practice to encapsulate these compounds to ensure their stability and controlled release in the final product [13]. In encapsulation, the core material is emulsified in the matrix of wall material, and then water is removed to obtain the dry product. Spray drying (SD) is a widely used encapsulation technique for flavors and oils with its compatibility with many wall materials, low processing cost, good retention of volatiles, and possibility of continuous operation [14,15,16,17,18]. In this technique, water is rapidly evaporated from atomized liquid particles with the help of hot air that blows into the chamber [19]. The type of atomization affects the final powder quality and product quality. Conventional nozzles, single-fluid or two fluids high-pressure spray nozzles are commonly used in the atomization of the feed in SD. However, the ultrasonic nozzle, one of the new atomization types, offers some advantages like uniform size distribution, good droplet sphericity, and protection of bioactive compounds [20]. Spray freeze-drying (SFD), with its low-temperature feature, is an alternative to SD in terms of preserving some heat-sensitive materials [21]. In SFD, firstly, fine frozen droplets are obtained with the atomization of feed into a cryogenic fluid, and then the water is removed by freeze-drying [16,22]. SFD is preferred to FD as it produces powders with more controlled particle size distributions [23].

Freeze-drying (FD) is another widely used encapsulation method in which water is removed from the frozen samples by sublimation. The freeze-dried products retain the initial quality characteristics well, such as nutrition, texture, and flavor, since there is no heat treatment. However, long drying times, higher energy consumption, and high process costs limit its industrial applications. The dry powders with a highly porous structure are important characteristics of powders obtained by both SFD and FD [24]. Although the porous wall structure is advantageous in terms of solubility and flowability, it could be disadvantageous in the case of long-lasting release of aroma [25]. In this study, three different encapsulation techniques were examined in terms of the encapsulation of the aroma compound of *Pistacia terebinthus* L. fruit oil. SD has been chosen due to the most used encapsulation technique. FD and SFD techniques carried out at low-temperature conditions have been chosen since the volatile compounds might be more retained at the end of the encapsulation process.

The choice of proper wall material is the first and most critical step affecting the final powder characteristics and encapsulation efficiency. There are a number of natural and synthetic polymers available as wall materials with their own advantages and disadvantages. Gum acacia (Arabic) has been addressed as an effective wall material for the encapsulation of oils and volatiles with high volatile retention due to its film-forming and emulsifying properties [26,27]. However, there are some challenges, such as limited availability, high cost, and impurity in gum acacia used in encapsulation. Therefore, researchers are looking for combinations of wall materials that provide good flavor retention by partially replacing gum acacia with another wall material. In this respect, a combination of gum acacia with maltodextrin, one of the commonly used wall materials, has been successfully used in encapsulation of 2-acetyl-1-pyrroline, a major flavor component of aromatic rice [28], pine flavor [29], Lippia sidoides essential oil [26], nutmeg essential oil [30], cinnamon essential oil [31], pterodon emarginatus essential oils [32], ethyl butyrate as the model flavor [33] and sulfur aroma compounds [34].

PTFO is a unique oil not only with its fatty acid composition but also with its aroma composition. However, the number of studies on the utilization of terebinth fruit oil is limited. It is essential to find alternative forms of this valuable oil to increase its use. In this respect, encapsulation of PTFO could expand its possible uses. Although there is only one study [35] on microencapsulation of PTFO, the study focuses on encapsulation efficiency and color characteristics of the powder. To the best of our knowledge, there is a lack of studies on the aroma composition of PTFO and the aroma retention of powders obtained by different encapsulation techniques. This is a significant issue to be studied because it can be a promising alternative for the food and cosmetic industry with a similar scent to pistachio nuts. Therefore, the objectives of this study were (1) to investigate the aroma composition of PTFO, (2) to investigate the effect of different MD:GA ratios on emulsion properties, and (3) to determine the effect of encapsulation techniques (SFD, SD, and FD) on powder characteristics and aroma retention.

## 2. Materials and Methods

### 2.1. Material and Reagents

*Pistacia terebinthus* L. fruits were collected during the ripening period (September to October) from Adıyaman, Turkey, and dried under the sun for two days (4.11 ± 0.08% wb). It was held at 4 °C until the oil extraction process. Maltodextrin (Roquette- Dextrose equivalent:6) and gum acacia (Alland & Robert, Paris, France) used in emulsion preparation were kindly obtained from a local company in Gaziantep, Turkey. All chemicals and reagents used in this study were of analytical, HPLC, or GC grade.

### 2.2. Methods

#### 2.2.1. Oil Extraction

The oil of dried fruits was extracted by cold pressing (hydraulic oil press, Ceselsan, YP 0420, Giresun, Turkey). The cold processing method was selected since it better preserves the taste, flavor, aroma, and nutritional value of the oil. Then, the oil was centrifuged at 10,000 rpm for 10 min to remove the precipitate. The obtained oil was packed immediately in amber-colored bottles and held at −18 °C till further experiments.

#### 2.2.2. Determination of Oil Characteristics

Peroxide value (determination of primary oxidation products), free fatty acid content (determination of hydrolytic rancidity level), p-anisidine value (determination of secondary oxidation products), and TBARS (determination of secondary oxidation products) are commonly used to report oil quality. Peroxide value was determined according to AOCS Methods (Cd 8-53) [36] and expressed as milliequivalents (meq) of O_2_ per kilogram of oil. Free fatty acid content was analyzed according to AOCS Cd 3d-63 [36]. Free fatty acids were expressed as % oleic acid. According to AOCS Cd 18-90, the p-anisidine value of PTFO was calculated [36]. The method of Baştürk et al. [37] was used to calculate the TBARS content of the oil.

The color was measured using Hunter-Lab ColorFlex (HunterLab, model A-60-1010-615, Reston, VA, USA) and expressed as L* (lightness), a* (red–green), and b* (yellow–blue) values. The fatty acid composition was obtained according to AOCS Official Method Ca 5a-40 [36]. Agilent GC-FID (GC Agilent 1100) was used in fatty acid identification and quantification.

#### 2.2.3. Preparation of Emulsion

Oil-in-water (*o*/*w*) emulsions were prepared by 10.0% (*w*/*w*) oil and 90.0% (*w*/*w*) of continuous aqueous phase. In the aqueous phase, different ratios of GA:MD (25:75, 50:50, 75:25) have been tried. The wall material contents were kept constant at 20%. Sodium azide was added to all emulsions at 0.01% to prevent microbial growth. It can inhibit and prevent the growth of microorganisms contaminated in various ways during the process. Firstly, the wall material combination was dissolved in distilled water by stirring at 300 rpm for 30 min at 80 °C, and then 10 g of PTFO was added to the solution. The mixture was homogenized with a homogenizer (model IKA, T18 Digital Ultra-Turrax) at 25,000 rpm for 5 min. The composition of emulsions is summarized in Table 1.

#### 2.2.4. Emulsion Characteristic Analysis

##### Creaming Stability

The creaming stability of emulsions was determined according to the method proposed by Zarena et al. [38]. First, 10 mL of emulsion was taken into a graduated cylinder. The serum separation was observed at room temperature for a week and measured daily. It was expressed as a percentage of the volume of the creaming part (*V_c_*) to the total volume of the emulsion (*V_t_*), as given in the following equation.

(1) $$Creaming\;\left(\%\right)=\frac{V_{c}}{V_{t}}\times 100$$

##### Droplet Size Measurement

A laser light scattering instrument (Mastersizer 3000, Malvern, Worcestershire, UK) was used to determine the mean particle size analysis of emulsions. A drop of the freshly prepared emulsion was supplied to the vessel in a device and dispersed in water using a magnetic stirrer. The average droplet sizes were obtained as volume mean diameter, d_43_, defined by the following equation.

(2) $$DeBroukerre\;Mean:D\left[4,3\right]=\sum N_{i}D_{i4}/N_{i}D_{i3}$$

where *D_i_* value is the geometric mean (the square root of upper*lower diameters) of diameters, and *N_i_* is the number of particles in the emulsion with diameter *D_i_*.

##### Microstructure Observation

The emulsions were observed by using polarized light microscopy (PLM) (Olymous BX 51 Model, Tokyo, Japan). One drop of emulsion was taken on a slide and covered with a coverslip. The microscopic images of emulsions were taken with a 40× magnification objective using a camera (PVC 100C, Pixera, Santa Clara, CA, USA). After this, images of the emulsion were taken.

#### 2.2.5. Encapsulation Methods

##### Spray Drying

A mini spray dryer (Büchi Labortechnik AG, Flawil, Switzerland) was used in spray drying experiments. The inlet air temperature, feed flow rate, air flow rate, and aspirator rate were set at 135 °C, 4 mL·min^−1^, 30 m^3^·h^−1^, and 90%, respectively. An ultrasonic nozzle (SD-UN) (60 kHz frequency, 1–15 W power output at nozzle, Buchi Corporation, Flawil, Switzerland) and a 2-fluidized nozzle (SD-2FD) were tried for atomization in spray drying experiments. The power of the ultrasonic nozzle was set at 9 W. The powder products were collected from the powder collector and stored in an amber-colored bottle at −18 °C until aroma and powder characteristic analysis.

##### Spray Freeze-Drying

Spray freeze-drying experiments were performed as described by Elik, Yanık and Göğüş [16]. An ultrasonic nozzle (60 kHz frequency, 1–15 W power output at nozzle, Buchi Corporation, Flawil, Switzerland) was used for atomization. Liquid nitrogen was used as cryogenic liquid. The feed flow rate was kept at 4 mL·min^−1^ and atomization power for the ultrasonic nozzle was set at 9 W. After removing the excess liquid nitrogen, the frozen droplets were transferred to a freeze dryer (CHRIST, Alpha 1-4 LSC, Osterode am Harz, Germany) to sublimate water. The powder obtained was packed in an amber-colored bottle and stored at −18 °C until aroma and powder characteristic analysis.

##### Freeze-Drying

Freeze-drying was performed using a freeze dryer (CHRIST, Alpha 1-4 LSC). Emulsion frozen at −70 °C was placed in a freeze dryer. The frozen sample was dried at −45 °C for 2 days. The freeze-dried product was packed in an amber-colored bottle and stored at −18 °C until aroma and powder characteristic analysis.

#### 2.2.6. Encapsulation Efficiency and Drying Yield

First of all the surface oil content was determined as described by Quispe-Condori et al. [39]. Shortly after, surface oil weight was calculated from the weight loss after repeated hexane wash of the encapsulated powder. Then, the percentage of encapsulated oil was expressed as percentage of encapsulated oil to total oil weight in the powder, as given in the following equation.

(3) $$Encapsulation\;efficiency\;\left(\%\right)=\frac{Total\;Oil-Surface\;Oil}{Total\;Oil}\times 100$$

Drying yield was calculated as the percentage of total dry mass of obtained powder (*PDM*) to the total dry matter amount in the feed (*FDM*), as given below.

(4) $$Drying\;yield\;\left(\%\right)=\frac{PDM}{FDM}\times 100$$

#### 2.2.7. Determination of Bulk and Tapped Density and Flowability Characteristics

The method proposed by Carneiro et al. [40] was used to determine the bulk density (ρ0) and tapped density (ρ50) of powder samples. Briefly, a graduated cylinder was filled with 3 g of the encapsulated powder and its initial volume was recorded. Then, the final volume was recorded after tapping the cylinder 50 times. Bulk density and tapped density were calculated as a mass-to-volume ratio. Bulk and tapped densities were measured in triplicate and the average was given as the result. The flow properties of the powder product, Carr’s index (CI), and Hausner ratio (HR) were calculated as described by Jinapong et al. [41] and calculated using the following equations.

HR = ρ50/ρ0     CI = (ρ50 − ρ0/ρ50) × 100(5)

#### 2.2.8. Moisture Content

The moisture content of powders was determined by vacuum drying at 70 °C. The percentage of weight loss was expressed as moisture content. Moisture content determination was repeated 3 times and the average was given as result.

#### 2.2.9. Analysis of Aroma Compounds

##### Headspace Solid-Phase Microextraction (HS-SPME) for Oil

Extraction of the volatile compounds from terebinth fruit oil was performed with some modification using headspace solid-phase microextraction (HS-SPME) methods described by Jeleń et al. [42] and Zhu et al. [43]. Approximately 3 g of oil was taken into a 20 mL glass vial with 1 μL of 4-nonanol as the internal standard. Then, it was agitated at 500 rpm to equilibrate at 60 °C for 45 min. SPME fibre coated with (50/30 mm DVB/Carboxen/PDMS, 1 cm, Supelco, Bellefonte, PA) was used as a sorbent. After extraction, fiber was removed from the vial and immediately transferred to the injection port of GC–MS to thermally desorb the analytes at 250 °C for 5 min.

##### Headspace Solid-Phase Microextraction (HS-SPME) for Powder

The method proposed by Vukoja et al. [44] was followed to extract volatile compounds from the powder samples using HS-SPME. Simply, 3 g of sample was weighed into a 20 mL glass vial with 1 μL of 4-nonanol as the internal standard, followed by the addition of 5 mL saline solution (25%). It was equilibrated and desorbed, as described above for the oil.

##### GC–MS Analysis of Aroma Compounds

GC–MS analysis was conducted as previously described by Zhu, Healy, Sevindik, Sun, Selli, Kelebek and Tiwari [43] using GC (Agilent 6890) equipped with a flame ionization detector (FID) and a mass selective detector (Agilent 5973-MSD). Aroma compounds were separated on a DB-Wax column (30 m length × 0.25 mm i.d. × 0.5 μm thickness, CA, USA). Helium was used as the carrier gas at a flow rate of 1.5 mL·min^−1^. The temperature of the FID detector and injector were set at 280 and 270 °C, respectively. The oven temperature is programmed as follows: initially the temperature was held at 40 °C for 4 min, increased to 90 °C at 3 °C/min, to 130 °C at 4 °C/min, and finally to 240 °C. 5 °C/min and held at this temperature for 8 min. Mass spectra were obtained in an electronic impact mode of 70 eV with a mass range of 30–300 amu. Identification of all volatile compounds was confirmed by matching the mass spectra obtained with the mass spectra available in the NIST database and the injection of standard compounds. The experimentally determined linear retention index (LRI) by Kovats was also compared with the RI in NIST WebBook and literature. The quantification of identified volatile compounds was made by utilizing the internal standard (4-nonanol). Two repetitions were conducted for each sample. After the quantification, the aroma retention (%) for aroma compounds (α-pinene, sabinene, β-myrcene, (Z)-β-ocimene, ocimene and linalool) were calculated on the basis of oil according to the formula given below.

(6) $$\%\;Retention=\frac{Concentration\;in\;the\;total\;oil\;in\;powder\;(\frac{\mu g}{kgoil})}{Concentration\;in\;\;initial\;oil\;load\;(\frac{\mu g}{kgoil})}\times 100$$

### 2.3. Statistical Analysis

The results were expressed as a mean ± standard deviation (SD) of three measurements. The significance between the data obtained for emulsion and powder characteristics was statistically analyzed by using one-way analysis of variance (ANOVA) and compared by Duncan’s multiple range tests using the SPSS software package program (SPSS Inc., Chicago, IL, USA-Version 25). The results were evaluated at a *p* < 0.05 significance level.

## 3. Results and Discussion

### 3.1. Pistacia terebinth L. Fruit Oil Quality Parameters and Aroma Composition

Some physical and chemical properties of cold-pressed PTFO are shown in Table 2. The low acidity and peroxide value of PTFO showed that the prepared oil has low hydrolytic and oxidative rancidity. According to the results, PTFO is rich in unsaturated fatty acid and has a quite bright yellow color.

Table 3 demonstrates the aroma composition of terebinth fruit oil. In total, 24 different aroma compounds were identified. Terpenes were found to be the most abundant class, followed by ketone, acid, and ester compounds. The major aroma compounds were d-limonene (29,626 μg·kg^−1^ oil), ocimene (20,808 μg·kg^−1^ oil), α-terpinene (18,937 μg·kg^−1^ oil), and p-cymene (17,140 μg·kg^−1^ oil). Most of the aroma compounds identified in terebinth fruit oil were similar to those previously identified in the terebinth fruit by Amanpour, Guclu, Kelebek, and Selli [7]; Gogus, Ozel, Kocak, Hamilton, and Lewis [6]; and Özcan et al. [45]. 

Terpenes have a wide range of applications in food, cosmetics, and the pharmaceutical industry. Terpenoids, such as limonene, myrcene, α-pinene, and linalool, have been designated as generally recognized as safe (GRAS) by the FDA [46]. For instance, limonene has a lemon fragrance and is used as a flavoring agent in foods, beverages, chewing gum, ice cream, candy, baked goods, etc. [47]. Limonene also has functions such as preservation, bacteriostasis, anti-inflammation, and anti-tumor activity, as well as acting as an expectorant, anti-asthmatic, and cholagogue [48]. On the other hand, both α-pinene and β-pinene have many commercial applications. They are commonly used as flavor compounds to produce herbal or earthy flavors in food [49]. α-Pinene has been used to treat respiratory tract infections for centuries. Furthermore, it plays a crucial role in the fragrance and flavor industry [50]. Myrcene is another popular food additive used as a flavoring agent with a high production volume of 58,076 kg for Europe and 1188 kg for the USA. β-Myrcene is used in the food and beverages industries as well as in products such as cosmetics, soaps, and detergents. β-Myrcene is an important starting material for the manufacturing of some other scents and flavors [51]. Terpenes are displayed as the major class of aroma compounds in terebinth fruit oil. The total amount of terpenes in fruit oil was 184 mg·kg^−1^, corresponding to 96.7% of total aroma. Similarly, the aroma composition of oils obtained from different parts of terebinth plants like shoots, flowers, and fruits was reported to consist of mostly monoterpenic hydrocarbons, oxygenated monoterpenes, and sesquiterpenes [52,53]. The major volatiles detected were the same as those previously identified as the leading compounds in the terebinth seeds by Amanpour, Guclu, Kelebek, and Selli [7]. In the current study, major volatiles of terebinth oil were d-limonene (15.6%), ocimene (11%), α-terpinene (10%), p-cymene (9%), (Z)-β-ocimene (4.6%), and α-pinene (4.5%). Couladis et al. (2003) found that ripe fruit oil of Turkish terebinth contained limonene (32.0%), β-pinene (23%), myrcene (11%), α-pinene (5%), and terpinolene (7%). Also, Dhifi et al. [54] determined the major compounds of the essential oil of Tunisian terebinth seeds as β-pinene (38.3%), terpinene (12.8%), α-pinene (15.7%), p-cymene (15.6%), and limonene (0.2%). These differences may be a result of oil extraction, plant genetics, geographical location, climate, and environmental factors [45,54]. Other than terpenes, acetic acid and bornyl acetate were detected as minor volatiles, which was also reported in the terebinth seeds and oils in the literature [7,45,55]. Bornyl acetate, especially, was reported to be one of the major volatiles of terebinth oil samples collected from İzmir and Antalya, while it was not determined in the samples from the other cities of Turkey [45]. Again, this difference can be due to the factors mentioned above.

### 3.2. Comparison of Emulsion Characteristics

**Figure 1:**
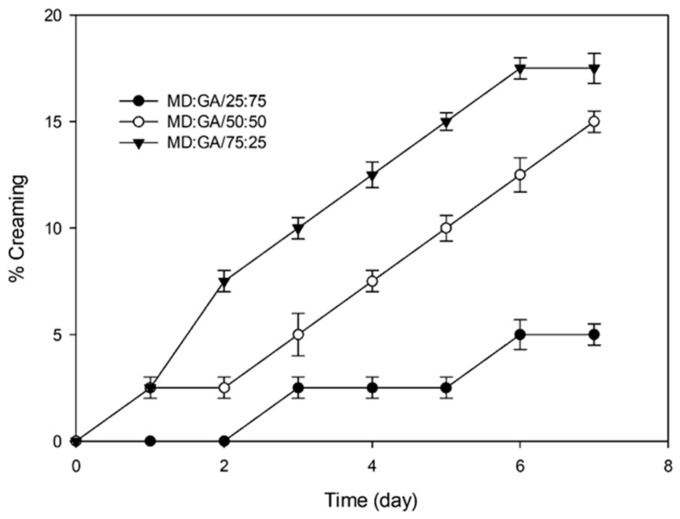
Creaming index of emulsions prepared in different MD:GA ratio.

Figure 1 shows the creaming index changes of emulsions prepared in different MD:GA ratios. Creaming is the most commonly encountered type of emulsion instability [56]. As seen in Figure 1, increasing the amount of GA in the emulsion has a positive effect on emulsion stability. At the end of 7 days of storage, the emulsion prepared in the ratio of 25:75 MD:GA was the most stable one with a 5% creaming index.

**Figure 2:**
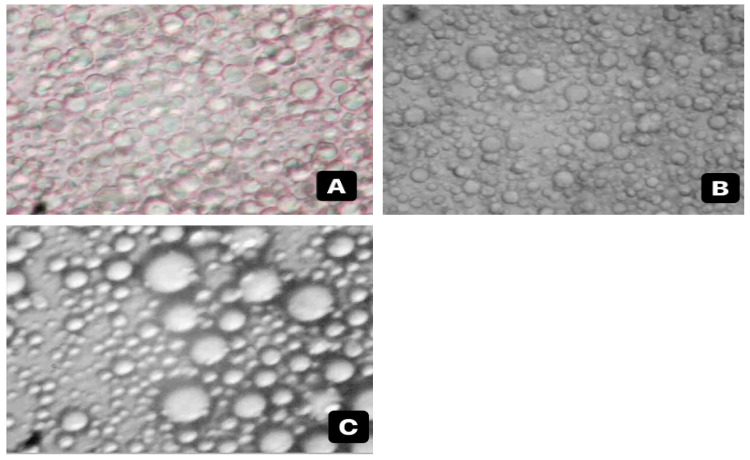
Microscopic images of emulsions obtained at 40× magnification (**A**) MD:GA/25:75 (**B**) MD:GA/50:50 (**C**) MD:GA/75:25.

Figure 2 shows the light microscopy images of emulsions prepared in different MD:GA ratios. The results support the creaming index results. The size and distribution of the droplets in the emulsion prepared with an MD:GA ratio of 25:75 was more uniform.

**Figure 3:**
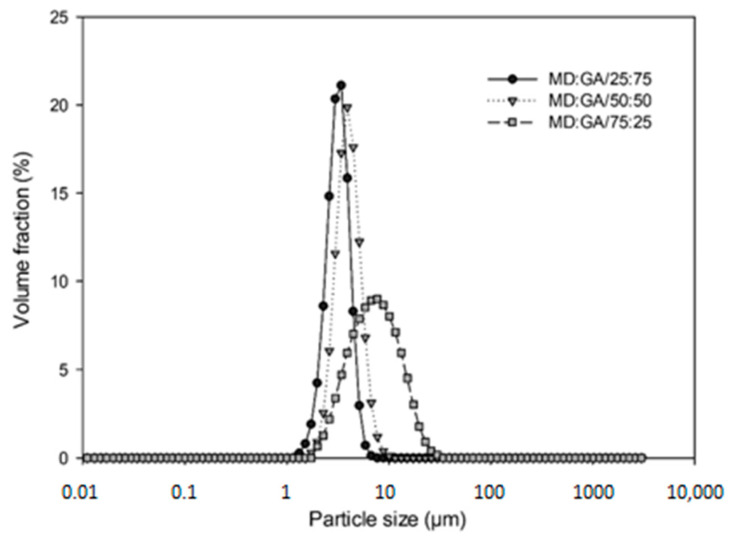
Particle size distribution graph of emulsions prepared in different MD:GA ratios.

The mean particle size (D[4,3]) values of emulsions prepared in MD:GA/25:75, MD:GA/50:50, and MD:GA/75:25 were 3.04 ± 0.03, 3.81 ± 0.02 and 13.52 ± 0.38, respectively. The volume distribution of droplets in emulsions prepared in different MD:GA ratios are given in Figure 3. The particle size distributions of the emulsion prepared by using MD:GA/25:75 and MD:GA/50:50 exhibited a monomodal distribution with a sharp peak, indicating a more uniform distribution, while MD:GA/75:25 showed a monomodal distribution and bell curve peak. The particle size of emulsions containing more MD shifted to larger particle sizes, resulting in lower stability. Particle size analysis and droplet size distribution results also show that MD:GA prepared in 75:25 has the smallest particle size and uniform distribution. One of the primary causes of emulsion stability loss is the increase in droplet size [57]. In encapsulation, an emulsion with reduced droplet size exhibits higher oil retention [27]. A stable emulsion is needed for increased encapsulation efficiency, relatively low surface oil, and higher volatile component retention [58]. For this consideration, the emulsion prepared in MD:GA ratio of 75:25 has been chosen for further encapsulation experiments.

### 3.3. Comparison of Encapsulation Methods in Terms of Efficiency, Yield and Powder Characteristics

Table 4 shows the effect of different encapsulation methods on encapsulation efficiency and drying yield. The encapsulation technique was found to have a significant effect (*p* < 0.05) on drying yield and encapsulation efficiency. Even if the drying yield was the highest for SFD, encapsulation efficiency was the lowest. Encapsulation efficiency is a measure of how successfully core material is encapsulated. Hence, lower surface oil on powder particles means better encapsulation efficiency [27]. The encapsulation efficiency of SD-2FN was found to be higher compared to SFD and SD-UN. This result obviously shows the superiority of the SD approach in the complete entrapment of the core material. Similarly, Elik, Yanık, and Göğüş [16] reported that encapsulation of carotenoid-enriched flaxseed oil using SD was better in terms of encapsulation efficiency than SFD. The encapsulation of PTFO using SD has been studied earlier by Poyrazoglu, Ozat, Coksari, Ozat, and Konar [35]. The best encapsulation efficiency in this study was 54.58% with inulin. This value is quite low compared to that obtained in the present study with MD:GA blend.

Bulk and tapped density of powders are important characteristics that affect packaging, transporting, storage, flowing properties, and storage stability. In this study, the bulk densities of powders obtained by using SFD, SD-UN, SD-2FN, and FD ranged between 0.19 and 0.29 g·ml^−1^ (Table 4). The bulk density of powder obtained by both SFD and FD was significantly lower (*p* < 0.05) than the SD ones. Closer bulk density values were earlier obtained in the encapsulation of vegetable oil (0.32–0.34 g·mL^−1^) [59], the production of soy milk powders (0.21–0.22 g·mL^−1^) [41], the microencapsulation of avocado oil (0.25–0.28 g·mL^−1^) [60], and the encapsulation of flaxseed oil (0.2113 g·mL^−1^) [16]. High bulk density means the low porosity between the particles, and so the amount of air occupied between the particles reduces. This would reduce the oxidation risk of powder and increase the storage stability [40].

The bulk and tapped densities should be close to each other for a powder with good flowability, so the Carr index will be small, and the Hausner Ratio will be close to 1.0 [61]. The compressibility or free-flowing quality is measured by CI, whereas the cohesiveness of powder is measured by HR. As shown in Table 4, no significant difference (*p* < 0.05) was detected between SFD, SD-UN, SD-2FN, and FD in terms of CI and HR. While CI for MD:GA/25:75 was determined to be in the range from 14.33 to 20.58, the HR was determined in the range of 1.16–1.25 using SFD, SD-UN, SD-2FN, and FD. “There are intermediate scales for CI between 11–15 or HR between 1.12–1.18 is considered ‘good’ flow, CI between 16–20 or HR between 1.19–1.25 is considered ‘fair’ flow” [62]. Hence, the results of this study reveal that the powder obtained by FD had good flowability characteristics while other powders exhibited ‘fair’ flowability.

Table 4 also shows the average particle sizes of powders encapsulated by SFD, SD-UN, and SD-2FN. A significant (*p* < 0.05) difference was found between encapsulates produced with different encapsulation methods. The freeze-dried encapsulates had the biggest droplet size among the others. Similarly, Brishti et al. [63] described that in comparison to other drying processes, such as spray drying and oven drying, freeze-drying produced the largest particle size of mung bean protein isolates.

### 3.4. Comparison of Retention of the Aroma Compounds during the Different Encapsulation Process

Since it will be time-consuming to compare the retention of each aroma compound present in terebinth fruit oil using different encapsulation techniques, some critical aroma compounds, α-pinene, sabinene, β-myrcene, (Z)-β-ocimene, ocimene, and linalool were selected for comparison in this study. These aroma compounds are present in high quantities and were previously addressed as the most powerful aroma-active compounds with their higher flavor dilution factors by Amanpour, Guclu, Kelebek, and Selli [7]. Table 5 shows the percentage of retention of selected volatiles in encapsulates as a function of different encapsulation methods. Many factors, such as the ability of aroma compounds to form a complex with wall material, physicochemical properties of the volatile compounds, wall material type, and concentration, affect the retention and stability of volatile compounds [64]. In general, volatiles with high molecular weight, low vapor pressure, and low hydrophobicity have better retention in the carbohydrate-based matrix [44,65]. The target aroma compounds identified in this study were all in the terpene class with the same molecular weight (MW = 136.23) except linalool (MW = 154.25). Additionally, the same wall material (MD:GA of 25:75) has been used in all encapsulation methods. Hence, the hydrophobicity and vapor pressure of the compounds would be the main reasons for the retention of each aroma compound. In SD, the α-pinene and linalool are the most retained components, with almost 100% among the other ones. This result could be because linalool had the highest molecular weight and lowest vapor pressure among the target volatiles. On the other hand, although α-pinene had the highest vapor pressure, it showed the highest retention owing to its hydrophobic characteristics.

Aroma retention results were also consistent with encapsulation efficiency results. As was expected, aroma retention was quite low for SFD and FD methods due to their low encapsulation efficiency. The lower the encapsulation efficiency, the higher the amount of surface oil. This means they will be susceptible to more volatile loss [66]. In addition, the lower percent retention values of aroma compounds for the SFD method could be caused by the evaporation of volatiles in direct contact with liquid nitrogen in an open vessel. With the exception of β-myrcene, SD-UN resulted in the best aroma retention for all other target aroma compounds. The total retention of selected aroma compounds in SD-UN was the highest (73.52%), subsequently followed by SD-2FN (62.28%), FD (24.52%), and SFD (14.16%). Badee et al. [67] also reported that spray drying quickly removes water by vaporization from oil-in-water emulsions and provides high retention of volatiles. Similarly, Chen et al. [68] have found better retention of limonene in encapsulates obtained by SD than that obtained by FD. Interestingly, the retention of the β-myrcene was the highest (96.30%) in FD when it was almost at the same level (43–45%) for all other methods. Similarly, a study conducted to encapsulate the lemongrass essential oil by freeze-drying displayed that the highest retention obtained was 81.6 ± 5.8% for β−myrcene with the slow-tested freezing protocol and a mixture of 50% GA, 40% MD, and 10% XG (volume) [69]. Additionally, Bertolini et al. [70] reported that the encapsulation efficiency of spray drying using gum arabic was 74–88% for β−myrcene and 44–64% for linalool. The authors displayed that steric factors and chemical functionality (diffusion through the forming matrix and solubility) of volatile compounds are the factors influencing the retention of volatile monoterpenes in gum arabic capsules.

## 4. Conclusions

In the first stage of the encapsulation process, the most stable emulsion with PTFO was obtained at MD:GA ratio of 25:75. Encapsulation efficiency was the highest in SD-2FN (>95%). All powder samples exhibited good flowability. Limonene (29,626 μg·kg^−1^ oil), ocimene (20,808 μg·kg^−1^ oil), and α-terpinene (18,937 μg·kg^−1^ oil) were the main aroma compounds of cold-pressed terebinth fruit oil. SD-UN application resulted in the highest total volatile retention. The aroma compounds, particularly α-pinene, and linalool that characterized the PTFO, were successfully retained in MD:GA matrix in a ratio of 25:75 by using SD-UN. The findings of this study are promising in terms of obtaining pistachio nut-like odor for the food and cosmetic industry. On the other hand, future research should focus on the application of encapsulated PTFO on a food, cosmetic, or pharmaceutical formulation to evaluate its performance in a real system.

## Figures and Tables

**Table 1:** The composition of maltodextrin, gum Arabic, and PTFO in emulsions.

MD:GA Ratio	Maltodextrin (g)	Gum Acacia (g)	Water (g)	Oil (g)
25:75	5	15	70	10
50:50	10	10	70	10
75:25	15	5	70	10

**Table 2:** Physical and chemical properties of PTFO.

Physical-Chemical Parameters	Value
Acidity (oleic %)	1.11 ± 0.01
Peroxide (meq/kg oil)	2.52 ± 0.07
p-Anisidine value	not detected
TBARS	not detected
Color	
L*	77.33 ± 0.08
a*	16.71 ± 0.10
b*	97.74 ± 0.25
Fatty acid composition (%)	
Myristic acid (C14:0)	0.05 ± 0.03
Palmitic Acid (C16:0)	25.04 ± 0.21
Palmitoleic acid (C16:1)	5.12 ± 0.10
Heptadecanoic acid (C17:0)	0.01 ± 0.01
Margaric acid (C17:1)	0.1 ± 0.02
Stearic acid (C18:0)	1.58 ± 0.32
Oleic acid (C18:1)	51.77 ± 0.17
Linoleic acid (C18:2)	15.17 ± 0.40
Eicosanoic acid (C20:0)	0.11 ± 0.02
Linolenic acid (C18:3)	0.92 ± 0.03
Docosahexaenoic acid (C22:0)	0.02 ± 0.03
Eicosadienoic acid (C20:2)	0.01 ± 0.02
Lignoceric acid (C24:0)	0.04 ± 0.01

**Table 3:** Aroma composition of terebinth fruit oil.

LRI ^a^	Compounds	Concentration (μg·kg^−1^) ^b^	Identification ^c^
1023	*α*-Pinene	8588 ± 54.0	LRI, MS, Std
1112	*β*-Pinene	7778 ± 2.1	LRI, MS, Std
1119	Sabinene	4427 ± 21.8	LRI, MS, tent
1132	*p*-Xylene	15,321 ± 41.1	LRI, MS, Std
1144	*δ*-3-Carene	5241 ± 44.0	LRI, MS, tent
1160	*β*-Myrcene	1278 ± 19.0	LRI, MS, Std
1179	*α*-Terpinene	18,937 ± 27.6	LRI, MS, Std
1197	D-Limonene	29,626 ± 26.6	LRI, MS, Std
1210	*β*-Phellandrene	7220 ± 23.6	LRI, MS, Std
1254	*(Z)-β*-Ocimene	8685 ± 225.0	LRI, MS, Std
1236	Ocimene	20,808 ± 146.0	LRI, MS, Std
1268	*p*-Cymene	17,140 ± 11.4	LRI, MS, Std
1286	*α*-Terpinolene	14,880 ± 77.2	LRI, MS, tent
1366	Alloocimene	11,545 ± 46.1	LRI, MS, tent
1441	Acetic acid	2678 ± 2.1	LRI, MS, Std
1486	*α*-Copaene	1567 ± 47.0	LRI, MS, Std
1545	Linalool	1984 ± 69.2	LRI, MS, Std
1574	Bornyl acetate	1287 ± 57.7	LRI, MS, Std
1596	4-Terpineol	1352 ± 56.7	LRI, MS, Std
1668	*α*-Terpineol	1562 ± 41.9	LRI, MS, Std
1711	Piperitone	2232 ± 51.5	LRI, MS, tent
1749	*δ*-Cadinene	1884 ± 22.7	LRI, MS, Std
1832	*(-)*-Calamenene	3123 ± 37.2	LRI, MS, tent
2116	Thymol	644 ± 20.0	LRI, MS, Std
	General Total	189,785 ± 248.0	

^a^ Linear retention index calculated on the DB-WAX capillary column. ^b^ Concentration: Results are the means of three repetitions as μg/kg. ^c^ Identification: Methods of identification: LRI (linear retention index), MS tent (tentatively identified by MS), Std (chemical standard).

**Table 4:** The effect of drying method on encapsulation efficiency, drying yield, and powder characteristics.

	Encapsulation Methods			
	SFD	SD-2FN	SD-UN	FD
Encapsulation efficiency (%)	51.66 ±4.16 ^a^	96.33 ±2.08 ^b^	93.33 ±2.30 ^b^	63.00 ± 3.61 ^c^
Drying yield (%)	84.40 ± 1.75 ^a^	74.27 ± 1.05 ^b^	45.27 ± 1.80 ^c^	95.39 ±0.82 ^d^
Powder Properties				
Moisture content (%)	0.23 ± 0.01 ^a^	1.27 ± 0.59 ^b^	1.31 ± 0.27 ^b^	0.57 ± 0.22 ^a^
Bulk density (g·mL^−1^)	0.19 ± 0.01 ^a^	0.29 ± 0.01 ^b^	0.27 ± 0.02 ^b^	0.20 ± 0.01 ^a^
Tapped density (g·mL^−1^)	0.24 ± 0.01 ^a^	0.35 ± 0.01 ^b^	0.33 ± 0.02 ^b^	0.24 ± 0.01 ^a^
Carr index	20.58 ± 1.00 ^a^	18.68 ± 4.28 ^ab^	17.87 ± 3.13 ^ab^	14.33 ± 1.52 ^b^
Hausner ratio	1.25 ± 0.01 ^a^	1.22 ± 0.06 ^ab^	1.21 ± 0.05 ^ab^	1.16 ± 0.02 ^b^
Particle size D_43_ (μm)	3.18 ± 0.18 ^a^	11.62 ± 0.35 ^b^	6.64 ± 0.35 ^c^	14.22 ± 0.79 ^d^

All the given values are means of three (*n* = 3) determinations ± SD. ^abcd^ Means within a row with different letters are significantly different (*p* < 0.05). SFD, Spray Freeze Drying. SD-2FN, Spray Drying- 2 Fluid Nozzle. SD-UN, Spray Drying -Ultrasonic Nozzle. FD, Freeze Drying.

**Table 5:** Aroma retention percentages of selected aroma compounds in terebinth fruit oil by different encapsulation methods.

Compounds	Retention (%)			
	SFD	FD	SD-2FN	SD-UN
*α*-Pinene	42.42 ± 0.37 ^a^	57.64 ± 1.24 ^b^	90.31 ± 2.19 ^c^	104.06 ± 3.47 ^d^
Sabinene	8.90 ± 0.29 ^a^	11.75 ± 0.32 ^b^	39.58 ± 1.27 ^c^	46.07 ± 1.49 ^d^
*β*-Myrcene	43.14 ± 0.39 ^a^	96.30 ± 3.51 ^b^	44.19 ± 1.34 ^a^	45.14 ± 0.89 ^a^
*(Z)-β*-Ocimene	5.87 ± 0.21 ^a^	14.08 ± 0.90 ^b^	61.13 ± 0,57 ^c^	31.95 ± 0.41 ^d^
Ocimene	5.32 ± 0.12 ^a^	13.84 ± 0.76 ^b^	52.58 ± 0.45 ^c^	82.28 ± 1.14 ^d^
Linalool	13.80 ± 0.46 ^a^	22.17 ± 0.66 ^b^	97.32 ± 0,8 ^c^	99.71 ± 1.86 ^d^
Total	14.16 ± 0.13 ^a^	24.52 ± 0.17 ^b^	62.28 ± 0.1 ^c^	73.52 ± 0.63 ^d^

^abcd^ Means within a row with different letters are significantly different (*p* < 0.05).

## Data Availability

The data used to support the findings of this study can be made available by the corresponding author upon request.
